# Beards

**Published:** 1874-02

**Authors:** 


### BEARDS.

THE wise and kind Infinite never made any thing in vain. Every created thing has not only its use, but its uses. Wearing the beard is no exception to the universal law. The beard was first mentioned thirty-three centuries ago, in connection with a Mosaic injunction, that it should not be "marred"—deformed. Its first great design, perhaps, was to distinguish the sexes, to inspire personal dignity, self-respect, and the deference of woman. The next great use is its influence in the preservation of man in those out-door exposures to winds, and cold, and dust, and accidents from which women are exempt, from its being more natural for her to remain in-doors in attention to domestic duties. Since we first mentioned, some five years ago, the advantages of keeping the mouth shut, as a preservative against colds, pleurisies, and pneumonias, by its sending the air to the lungs through the circuit of the head, thus warming it, a book has been written on the subject. The beard on the upper-lip is kept warm by its living connection with the body, and by the warm air constantly passing out of the nostrils; this warmth is imparted to the incoming air, and thus effectually prevents those dangerous shocks of cold driving in upon the warm lungs, which so often cut short human life in three or four days. The beard being warm, evaporates any dampness in the atmosphere, to a greater or less degree, and thus gives a purer air to the lungs; rendered still more pure by the dust, with which the air is always full, being detained in the meshes of the hair.

The throat and upper part of the chest are greatly exposed to cold; their imprudent exposure engenders some of the most fatal forms of disease, such as Bronchitis, Consumption, Diphtheria, and the like. The beard is an extraordinary protection against cold. The thinnest gossamer vail over the face will make the coldest winds endurable. Delicate and silken as the hair is, its protecting influence in keeping the scalp comfortably warm, is very impressively appreciated by those who have become bald.

Inconsistent as it may seem at first sight, the beard not only keeps the parts genially warm in winter, but by its evaporating influence, cools the parts wonderfully in the hottest weather, to say nothing of its breaking the force of the hot sun.

Another advantage of the beard is its power to break the force of blows and arrest the stroke of a cutting instrument against so vital and otherwise easily vulnerable a part as the throat. Many persons aggravate throat complaints by mufflers, wearing scarfs or extra covering about the neck; these do keep the throat warm, but in every change of position of the head or face, some part of the neck or throat is moved from the covering; the covering does not adapt itself to or follow the movement, hence the cold air rushes in upon that unprotected part and chills it; but the beard follows every motion of the head or face faithfully, and thus is the most perfect muffler that can possibly be devised. Nature's provisions can not be interfered with with impunity. The Orientals, who shave the head and wear the beard, suffer more from ophthalmia, an eye-disease, but have fine teeth. Europeans, who shave the beard and wear the hair, suffer but little from ophthalmia, but have very defective teeth; this last result may arise from the beard modifying the coldness of the air which passes into the mouth, thus keeping the temperature of the teeth more equal. The early Christian fathers denounced shaving as a violation of the law of God. The beard of John Mayo of Germany touched the ground when he stood upright. Steel-grinders, stone-cutters, engineers, firemen, and all others who work in dust, heat, or steam, should especially wear the beard. Daily shaving is an intolerable nuisance, a useless waste of time.

